# HPLC-MS Quantification of Multiple Tyrosine Kinase Inhibitors in Patients with Solid Tumors: Method Validation and Clinical Application

**DOI:** 10.3390/pharmaceutics18080923

**Published:** 2026-07-27

**Authors:** Juliane Staudinger, Marcel Kemper, Carolin Krekeler, Lea Reitnauer, Annalen Bleckmann, Georg Hempel

**Affiliations:** 1 Department of Clinical Pharmacy, Institute of Pharmaceutical and Medicinal Chemistry, University Muenster, 48149 Muenster, Germany; j.staudinger@uni-muenster.de; 2 Medical Clinic A, Hematology, Oncology, Hemostaseology and Pneumology, University Hospital Muenster, 48149 Muenster, Germany; marcel.kemper@ukmuenster.de (M.K.); carolin.krekeler@ukmuenster.de (C.K.); leaelisabeth.reitnauer@ukmuenster.de (L.R.); annalen.bleckmann@ukmuenster.de (A.B.); 3 West German Cancer Center, University Hospital Muenster, 48149 Muenster, Germany

**Keywords:** tyrosine kinase inhibitors, non-small cell lung cancer, therapeutic drug monitoring, volumetric absorptive microsampling, high-performance liquid chromatography–mass spectrometry, osimertinib

## Abstract

**Objectives**: A high-performance liquid chromatography (HPLC) with mass spectrometry (MS) detection method was developed to quantify several tyrosine kinase inhibitors (TKIs) and their relevant metabolites. This method is suitable for therapeutic drug monitoring (TDM) of alectinib, brigatinib, dabrafenib, lenvatinib, lorlatinib, osimertinib and trametinib in patients with solid tumors using volumetric absorptive microsampling (VAMS^®^). **Methods**: The HPLC-MS system contained four pumps, a Turboflow HTLC CycloneTM 1.0 × 50 mm solid phase extraction column for analyte enrichment, and a Kinetex 2.6 µm C18 100Å, 100 × 3.0 mm column for analyte separation. An acetonitrile–water gradient was used for the separation, and the ions generated by ESI (+)-ionization were detected in single-ion mode. This method was validated according to recent European Medicines Agency (EMA) and Food and Drug Administration (FDA) guidelines. **Results**: The accuracy and precision shown during method validation were within the acceptable limits for all analytes in plasma and whole blood. All analytes showed acceptable stability in both matrices for at least 28 days when stored at −21 °C. So far, 100 venous plasma and 94 capillary blood samples have been collected and analyzed. **Conclusions**: We developed a reliable method to quantify several TKIs from plasma and capillary blood, which is intended for TDM purposes in clinical practice.

## 1. Introduction

Tyrosine kinase inhibitors (TKIs) have broad clinical applications in solid tumors and are widely used in patients with advanced non-small cell lung cancer (NSCLC) harboring actionable genetic alterations (AGAs), as well as in differentiated thyroid carcinoma (DTC). In these settings, TKIs have substantially improved patient prognosis and survival [1,2]. In contrast to classical chemotherapy combinations, TKIs are administered orally in an outpatient setting at a standard dose daily for longer time periods. Adverse drug reactions (ADRs) that require dose reductions occur frequently. In many cases, the development of toxicities strongly correlates with the overall exposure, which is believed to be a marker for efficacy that has been confirmed for several TKIs [3]. Therapeutic drug monitoring (TDM) enables practitioners and clinicians to identify patients not reaching efficacious exposure or patients with an increased risk of ADRs when exceeding the safety threshold [4]. However, TDM for TKIs so far is not routinely implemented in treatment guidelines for DTC and NSCLC patients with AGA.

**Figure 1:**
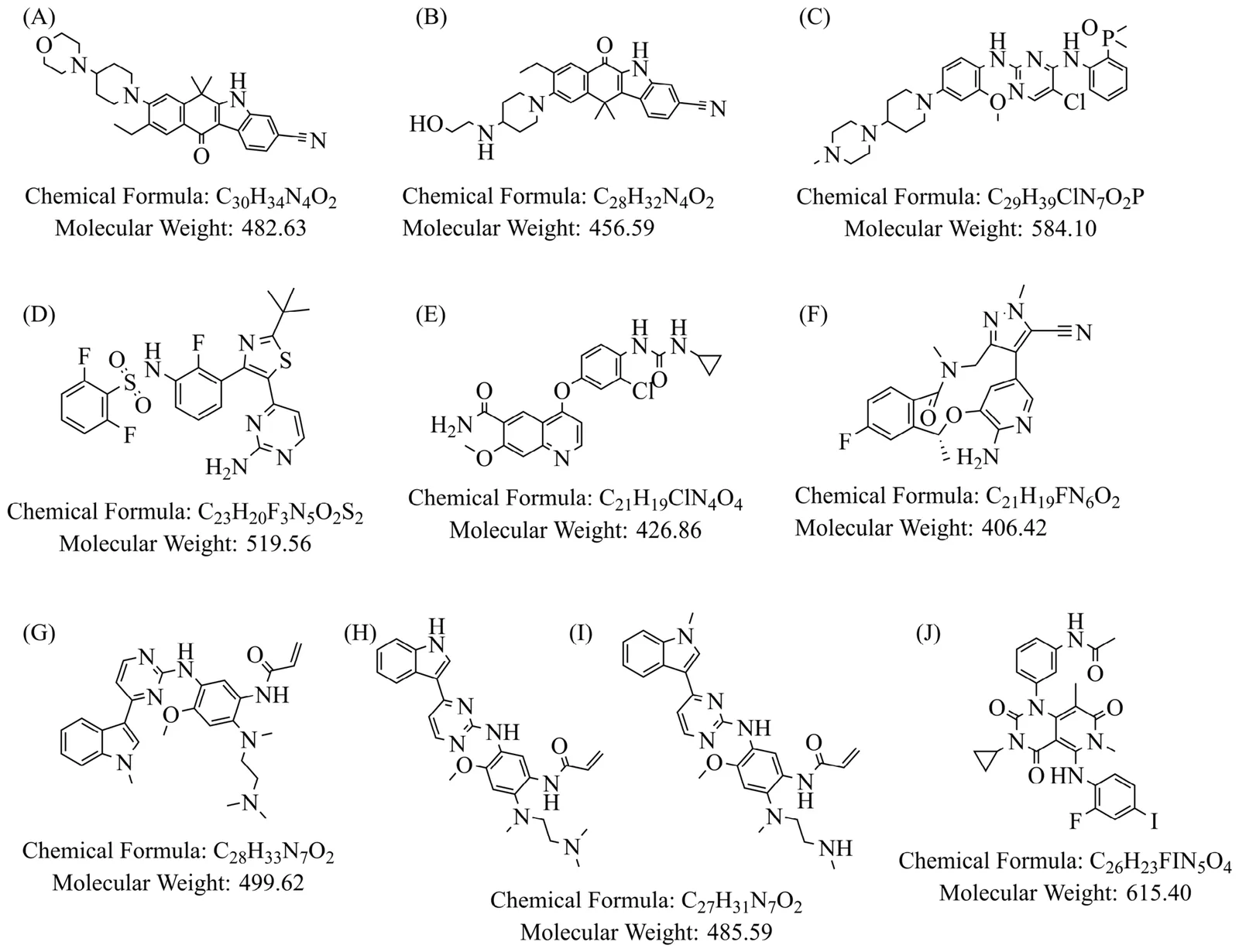
Structures, chemical formula and molecular weight [g/mol] for (**A**) alectinib, (**B**) alectinib-M4, (**C**) brigatinib, (**D**) dabrafenib, (**E**) lenvatinib, (**F**) lorlatinib, (**G**) osimertinib, (**H**) AZ-5104, (**I**) AZ-7550 and (**J**) trametinib.

Alectinib (ALE), brigatinib (BRI), dabrafenib (DAB), lorlatinib (LOR), osimertinib (OSI) and trametinib (TRA) are frequently used in NSCLC patients with AGA and lenvatinib (LEN) (Figure 1) in patients with radioiodine-refractory DTC. ALE shows an exposure–response relationship and LOR an exposure–toxicity relationship, whereas BRI and OSI show both. Results for LEN and TRA are inconclusive and need further investigation [3,5]. In addition to the parent compounds, both ALE and OSI are metabolized to pharmacologically active metabolites, and ALE-M4—the active metabolite of ALE—has not yet been evaluated with respect to its exposure–response relationship. In contrast, the active OSI metabolites AZ-5104 (OSI-M5) and AZ-7550 (OSI-M7) have been associated with exposure–toxicity relationships, which are attributed to their prolonged half-life and higher target affinity, respectively [6].

The simultaneous quantification of multiple TKIs has become an important approach in TDM routines to save time and improve efficiency. Rood et al. reported several methods determining concentrations of multiple drugs in plasma where LC-MS/MS was the predominantly used method for analyte detection because of its superior sensitivity and selectivity [7]. Another key factor in ensuring the feasibility of TDM is the selection of an appropriate sampling method and the minimization of the processing time. Microsampling techniques like dried blood spots (DBSs) and volumetric absorptive microsampling (VAMS^®^) showed promising results, for example, with at-home sampling. However, there are few methods reported, especially when focusing on the simultaneous determination of several analytes [8]. Examples by Meertens et al., Verougstraete and Stove, and Zimmermann et al. included an evaporation step during VAMS^®^ sample preparation for purification, which is a time-consuming step depending on the volume and the extracting agent [9,10,11].

This study primarily aims to establish a bioanalytical method within the framework of a TDM routine for ambulatory patients with solid tumors being treated with TKIs. In addition, the venipuncture as a standard sampling technique for TDM is being evaluated to lower patients’ burden. Capillary blood sampling is being assessed by practitioners and patients using the VAMS^®^ device Mitra^®^ with concomitant venipuncture. To determine the concentration from both matrices, an HPLC-MS method for ALE and its metabolite ALE-M4, BRI, DAB, LEN, LOR, OSI and its metabolites OSI-M5 and OSI-M7, and TRA has been developed based on the previous work of Opitz et al. [12].

## 2. Materials and Methods

### 2.1. Chemicals and Reagents

The analytes ALE, BRI, LOR, OSI-M5 and OSI-M7-mesylate were purchased from MedChem Express (Monmouth Junction, NJ, USA). ALE-M4, OSI and the internal standard 2H4-ALE-M4 were purchased from Toronto Research Chemicals Inc. (Toronto, ON, Canada). LEN-mesylate was purchased from Sigma-Aldrich Chemie GmbH (Taufkirchen, Germany). D9-DAB, 13C1-D3-OSI, and 13C6-TRA were obtained from Alsachim (Illkirch-Graffenstaden, France). Water was purified by a Milli-Q-Advantage A10 system by Merck (Darmstadt, Germany). Methanol (HPLC grade), acetonitrile (LC-MS grade), propan-2-ol (HPLC grade) and formic acid were obtained from Fisher Scientific U.K., Ltd. (Loughborough, UK) and an ammonia solution (≥25%) from Sigma-Aldrich Chemie GmbH (Taufkirchen, Germany). EDTA-anticoagulated whole blood and plasma were obtained from healthy volunteers at the blood donation service at the University Hospital Münster (Münster, Germany).

### 2.2. Software

The chemical structures were generated using ChemDraw 23.1.2 (revvity signals, Waltham, MA, USA). The graphs were created with R 4.4.2 utilizing RStudio 2026.05.1 (Posit Software, PBC, Boston, MA, USA). Chromatographic data were processed using LabSolutions version 5.60 SP2 (Shimadzu Corporation, Kyoto, Japan).

### 2.3. Equipment

The HPLC-system by Shimadzu Corporation (Kyoto, Japan) consisted of two degasser units DGU-20A5R, three LC-20ADXR pumps, one LC-20ADXR pump with a quaternary low pressure gradient system, an SIL-30AC autosampler, a CTO-20AC column oven and a CBM-20A control module, coupled with an LCMS2020 single quadrupole (SQ) mass spectrometer for detection. The chromatic separation and the on-line solid phase extraction were established using a Kinetex 2.6 µm C18 100 Å, 100 × 3.0 mm column from Phenomenex LTD (Aschaffenburg, Germany) and a TurboFlow^TM^ HTLC CycloneTM 1.0 × 50 mm solid phase extraction column from Thermo Fisher Scientific (Waltham, MA, USA), respectively. During the sample preparation, a Centrifuge 5430R and a Thermomixer comfort, both from Eppendorf SE (Hamburg, Germany), were used.

### 2.4. Chromatographic Conditions

An aliquot of 200 µL and 50 µL of the supernatant obtained from the VAMS^®^ and plasma, respectively, was injected and flushed onto the solid phase extraction column for purification. For the extraction, an aqueous ammonia solution (0.3%) and methanol (96/4 *v*/*v*) were used at 2.5 mL/min for the first 2 min. Afterwards, a mobile phase consisting of methanol, propan-2-ol and aqueous formic acid (45/45/10 *v*/*v*/*v*) with a flow of 0.8 mL/min was used for washing. The re-equilibration started after 20 min up to the end of the run.

For the analytical separation, the flow rate was 0.4 mL/min, the oven temperature was set at 40 °C and the mass spectrometer was in positive electrospray ionization mode. A gradient system was chosen with two mobile phases containing acetonitrile and water (A2: 5/95 and B2: 95/5), with 0.1% formic acid starting with 0% B2 for 2 min, increasing to 30% B2 within one minute, and finally increasing to 100% B2 between minute 6 and 16. The reconditioning started after 20 min, and the run ended at 25 min.

The analytes were detected in the selected ion monitoring mode with the *m*/*z* values: ALE 483.3, ALE-M4 457.3, BRI 584.1, DAB 520.1, LEN 427.1, LOR 407.0, OSI 500.3, OSI-M5 and OSI-M7 486.1, and TRA 616.1.

For ALE and ALE-M4, 2H4-ALE-M4 served as the internal standard (ISTD), and for DAB and TRA, the respective labeled forms D9-DAB and 13C6-TRA were used. The other analytes were quantified using 13C1-D3-OSI as the ISTD. The ISTDs were detected with the *m*/*z*-values: 2H4-ALE-M4 461.3, D9-DAB 529.1, 13C1-D3-OSI 504.2 and 13C6-TRA 622.1.

### 2.5. Stock Solution and Working Solution

For the stock solution, 1 mg of the free base or the equivalent of the salt of each analyte was dissolved in 1 mL DMSO and stored at −80 °C. For the working solution, the standards were combined and diluted with methanol to achieve a target concentration of 6.25 mg/L for OSI-M5 and OSI-M7 and 12.5 mg/L for the other analytes. Working solutions were stored at −21 °C. The working solution was then added to EDTA-anticoagulated whole blood (EDTA-WB) or plasma and diluted to obtain a calibration and quality control standards (Table 1). An amount of 50 µL of spiked plasma was processed further, and 10 µL of spiked EDTA-WB was absorbed by a VAMS^®^.

The stock solutions of the ISTDs ALE-M4-2H4, D9-DAB, 13C1-D3-OSI and 13C6-TRA were diluted 1:100 with methanol. An aliquot of 40 µL was added to 100 mL of acetonitrile, forming the extracting agent.

### 2.6. Sample Preparation

#### 2.6.1. Capillary Blood

The dried VAMS^®^ samples were placed in plastic tubes and re-wetted with 50 µL of double-distilled water. After 5 min, 250 µL of extracting agent was added, and the samples were shaken at 1400 rpm for 10 min, sonicated for 15 min, again shaken at 1400 rpm for 10 min and lastly centrifuged at 20,817 *g* at 8 °C for 10 min. The supernatant was transferred to an HPLC vial with a 350 µL insert.

#### 2.6.2. Plasma

An amount of 50 µL of plasma was mixed with 250 µL of the extracting agent, shaken for 10 min at 1400 rpm, and then centrifuged at 20,817 *g* at 8 °C for 10 min before the supernatant was transferred to an HPLC vial with a 350 µL insert.

### 2.7. Calibration and Calculation Procedure (Linearity)

Every measurement batch consisted of a blank sample, a zero sample (blank sample with ISTD), QCs and at least six calibration standards with the ranges and levels already described in Section 2.5, as specified in the EMA and FDA guidelines for bioanalytical methods [13,14]. To determine unknown samples, the concentration was plotted against the area ratio of the analyte and the corresponding ISTD, and a linear regression with the equation *y* = *mx* + *b* with 1/x^2^ weighting was applied.

### 2.8. Validation

The validation was performed according to EMA and FDA guidelines [13,14]. Deviating from the guidelines, only the stability suitable for the clinical study was tested. The hematocrit effect between VAMS^®^ and plasma was examined using clinical data in comparing patient samples.

#### 2.8.1. Accuracy and Precision

Accuracy and precision were calculated for both within- and between-run comparisons to determine the variation. For the within-run values, quintuplicates of each QC level were measured on one day, and for the between-run values, three duplicates of each QC level were examined on 3 separate days.

#### 2.8.2. Stability

To determine the stability, spiked EDTA-WB and plasma samples were prepared at all three QC levels and at the lower limit of quantification (LLOQ) and then stored at −21 °C until processing. On days 1, 7, 14, 21 and 28, the samples were analyzed and evaluated in relation to a freshly processed calibration series.

### 2.9. Patient Samples

Venous and capillary blood samples were obtained from patients with solid tumors during routine visits at the University Hospital Muenster, Germany. The capillary blood samples were collected using 10 µL VAMS^®^ obtained from Neoteryx (Torrance, CA, USA) by Trajan (Ringwood, VIC, Australia) according to the tutorial by Protti et al. [15]. VAMS^®^ samples were dried for 1 h under light exclusion and afterwards stored in a lightproof sealed plastic bag with 1 g of silica gel per VAMS^®^ at −21 °C. EDTA-WB was centrifuged at 239× *g* for 5 min at 8 °C to obtain plasma, which was immediately stored at −21 °C. The VAMS^®^ sample was taken shortly after the peripheral venous blood sample. The sample preparation for TKI measurement was carried out as specified above. Hematocrit values were obtained from routine measurements performed by the University Hospital Muenster. Written informed consent was given by all patients for blood sampling and data processing. The sample collection was conducted in accordance with the Declaration of Helsinki and approved by the Ethics Committee of the Chamber of Physicians Westfalen-Lippe and the University of Münster, Münster, Germany (Approval Code: 2023-124-f-S).

### 2.10. Hematocrit Influence on VAMS^®^-to-Plasma Ratio

To assess the influence of the hematocrit in patient samples, the VAMS^®^-to-plasma ratio (Equation (1)) was plotted against the individual hematocrit values, and a linear regression model was fitted to the respective data.

(1) $$Ratio=\frac{{concentration}_{VAMS}}{{concentration}_{Plasma}}$$

An in vivo hematocrit effect could be ruled out for the examined hematocrit range if the 95% confidence interval (95%-CI) of the slope of the linear regression includes 0. Samples below the lower limit of quantification were excluded from the analysis.

## 3. Results

**Figure 2:**
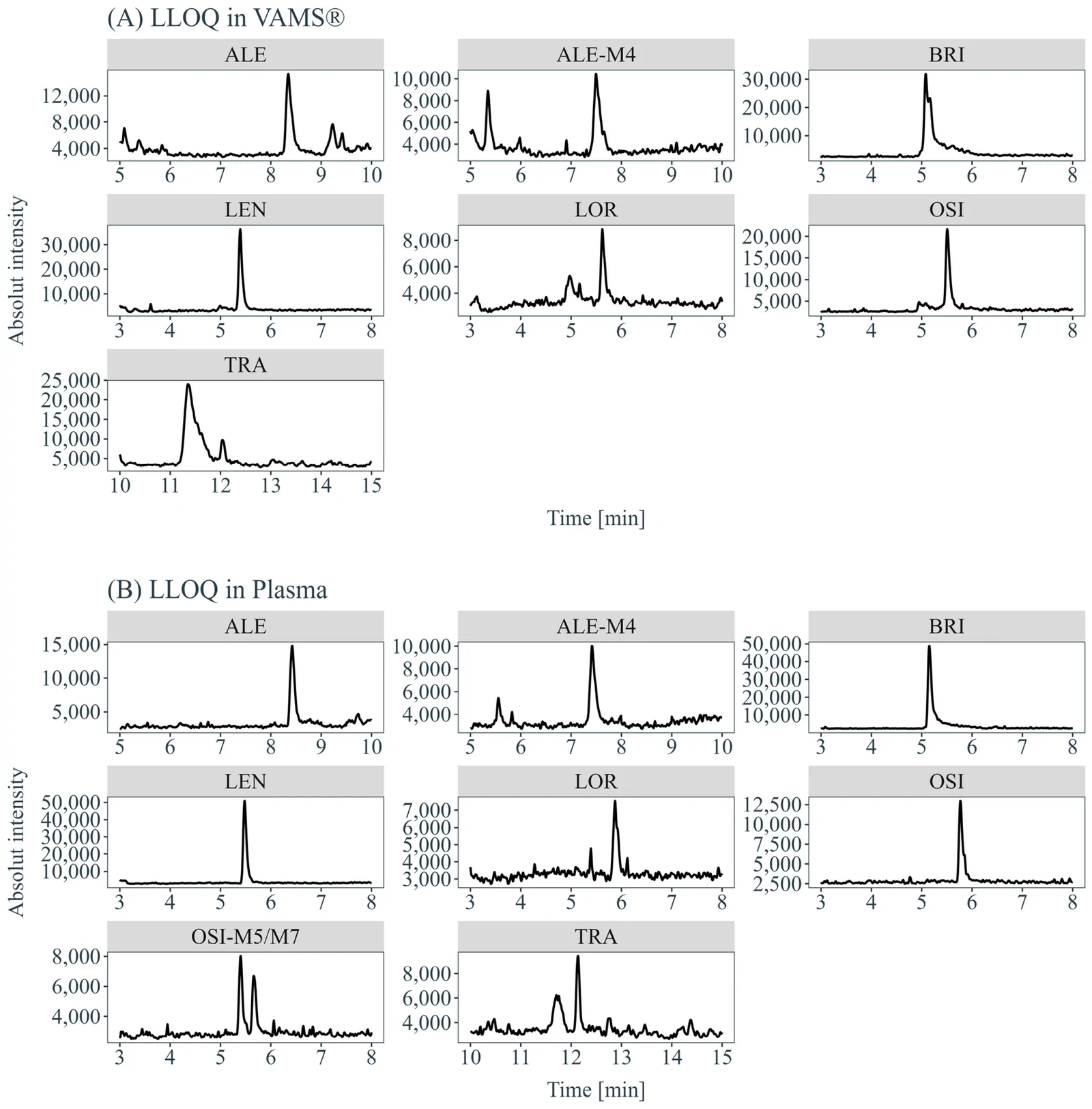
Chromatogram of (**A**) VAMS^®^ and (**B**) plasma samples.

To improve the feasibility of blood sampling in outpatient care, capillary blood was collected and transported using VAMS^®^. A sufficient sensitivity with a sample volume of only 10 µL was achieved by applying column switching, which enabled the injection of 200 µL of the extracted sample. The TurboFlow^TM^ column first removed endogenous matrix components from the biological sample matrix while retaining the analyte and the IS, resulting in a cleaner extract for subsequent separation on the analytical column. Chromatograms at the LLOQ are presented in Figure 2.

### 3.1. Linearity

Six calibration series in EDTA-WB and plasma were measured on six different days, showing accuracies within the permitted limits of ±20% for the LLOQ and ±15% for all other calibration points, respectively. The corresponding coefficients of determination (R2) were above 0.99 for all analytes in EDTA-whole blood and plasma (Table S1).

### 3.2. Selectivity

To examine selectivity, EDTA-WB and blank plasma samples from six different individuals were prepared and analyzed to rule out possible interferences of endogenous matrix components with the analyte peaks. The peak area ratio had to be less than 20% of the LLOQ’s area and less than 5% of the internal standard’s area. The area ratios of BRI, OSI, OSI-M5, and OSI-M7 ranged between 10% and 20%, whereas those of ALE, ALE-M4, and TRA lied between 1% and 10%, while DAB and LOR exhibited area ratios below 1%, showing adequate selectivity for all analytes. The ratios of the internal standards were below 5% (Table S2).

### 3.3. Recovery and Matrix Effect

The recovery was determined by comparing the peak area of the LQC, MQC and HQC prepared in the matrix with the area of blank matrix samples spiked after processing. To obtain the respective concentration, the working solution was diluted with acetonitrile. Then, 50 and 10 µL were added to the blank plasma and VAMS^®^ sample, respectively. The same volume of acetonitrile was added to the matrix QCs to compensate for the dilution. The recovery was then determined by Formula (2). Table 2 summarizes the values obtained for recovery.

(2) $$Recovery\;\left[\%\right]=\frac{{Area}_{Matrix\;QC-Sample}}{{Area}_{Spiked\;Blank-Sample}}\times 100\%$$

The recovery for plasma samples was above 85% for all analytes and concentration levels. The consistency was very high, as the coefficient of variation was below 10%. The variation in the recovery was higher for the VAMS^®^ samples. However, the coefficient of variation did not surpass 15%, except for TRA at LQC, where one VAMS^®^ sample did not fill correctly leading to a strong reduction in the signal; therefore, it had a low recovery not meeting the specifications. OSI and its metabolites showed the lowest recovery ranging from 56.6 to 80.3%.

To determine the extent of the matrix effect, triplicates of spiked acetonitrile (neat sample) were measured at LQC, MQC and HQC. The matrix effect was then determined based on the method proposed by Matuszewski et al. using Formula (3) [16].

(3) $$Matrix\;Effect\;\left[\%\right]=\frac{{Area}_{Spiked\;blank-Sample}}{{Area}_{Neat\;Sample}}\times 100\%$$

Matrix effects above 100% indicate ion enhancement, whereas values below 85% indicate ion suppression. In plasma, LOR showed large ion suppression and OSI-M7 showed significant ion enhancement, with matrix effects ranging from 61.7 to 92.5% and 123 to 153%, respectively. All analytes showed high ion enhancement in VAMS^®^, ranging from 106 to 218% with low variability, as reflected by the coefficients of variation not exceeding 15% (Table 2).

### 3.4. Accuracy and Precision

The accuracy and precision (expressed as the coefficient of variation (CV%)) for the EDTA-WB and plasma samples were determined in one within-run comparison and six between-runs comparisons performed on at least three days. Results are shown in Table 3 and are within the allowed limit of ±15%.

### 3.5. Carry-Over

During the assessment of the carry-over, three blanks were measured after the measurement of an upper limit of quantification (ULOQ) sample on three different occasions. The results are summarized in Table S3 and are within the expected limits for DAB, LOR and TRA. Exceeding the limit of 20% of the LLOQ’s area in the first blank after the ULOQ are ALE, ALE-M4, BRI, OSI, OSI-M5 and OSI-M7.

### 3.6. Stability

**Figure 3:**
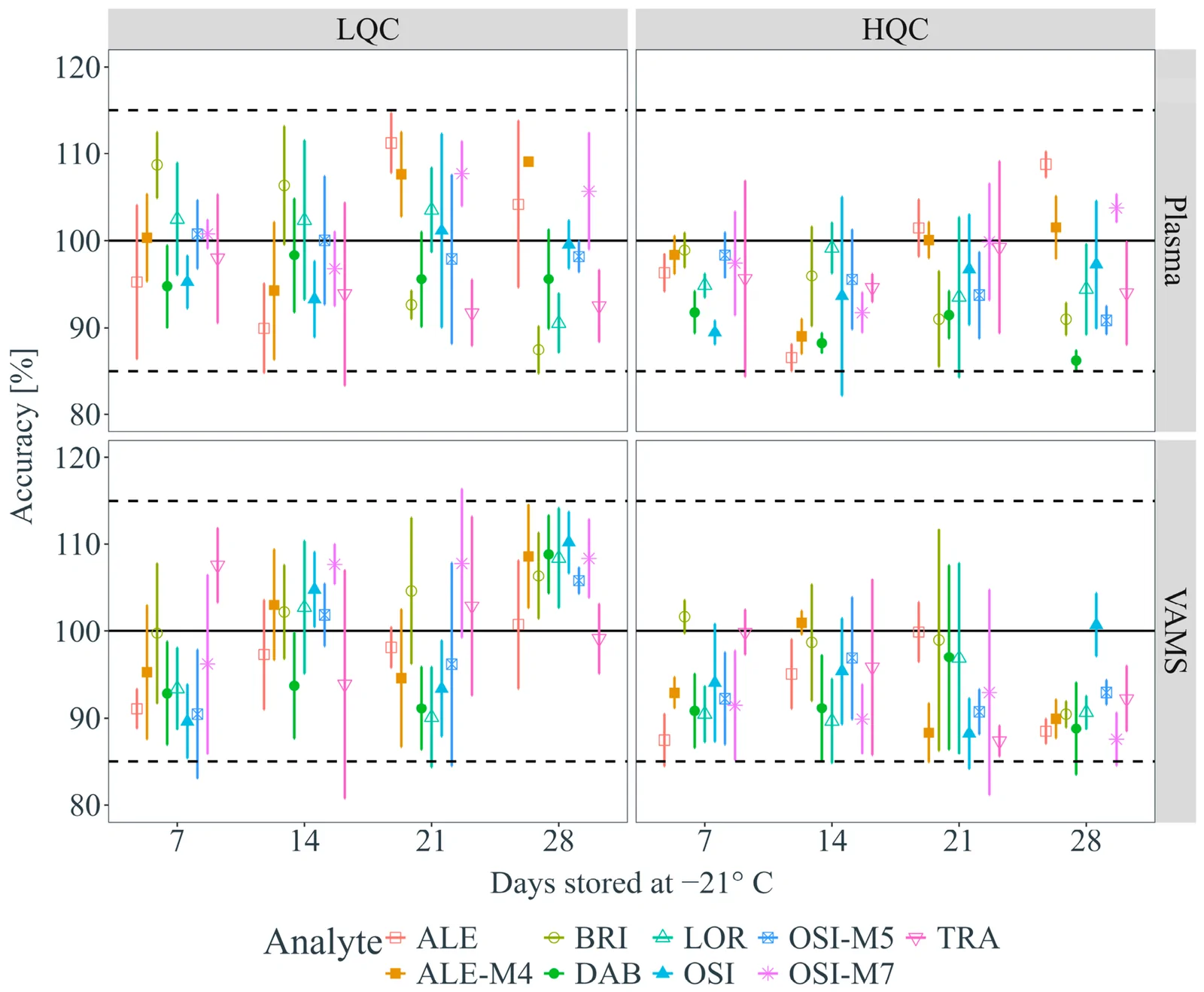
Stability of plasma and VAMS^®^ samples at LQC and HQC stored at −21 °C, with mean accuracy and standard deviation displayed per analyte and matrix over time. The dashed lines represent the ±15% limits around the ideal accuracy value of 100%, which is shown as the solid black line.

To test the stability suitable for this study, triplicates of LQC and HQC were prepared in EDTA-WB and plasma and stored at −21 °C for 7, 14, 21 and 28 days. On the corresponding days, a calibration curve was freshly processed and analyzed. All samples in both matrices were within the limit of ±15%, as shown in Figure 3, over the tested period of time.

### 3.7. Reinjection Reproducibility

Quality control samples were prepared in plasma and stored in the autosampler at +8°. Five replicates of LQC, MQC and HQC were measured after 12 h. All replicates for each analyte met the criteria of ±15% at all three levels (Table S4).

### 3.8. Dilution Integrity

Two- and fourfold samples of the HQC were prepared in plasma and diluted 1:1 and 1:3, respectively, and a blank matrix and five replicates of each were measured. All measured concentrations were within the limit of ±15% of the expected concentration (Table S5).

### 3.9. Patient Samples

**Figure 4:**
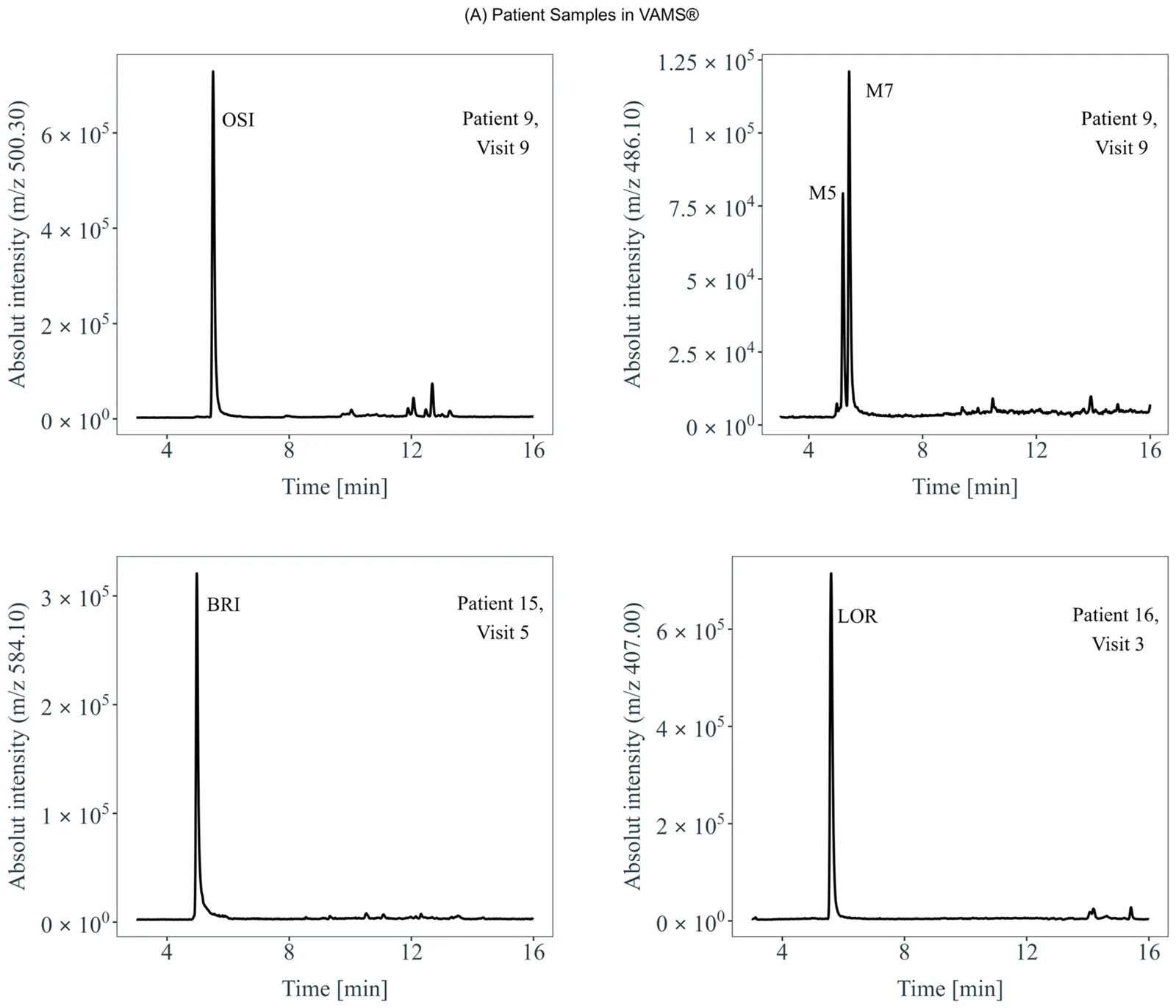

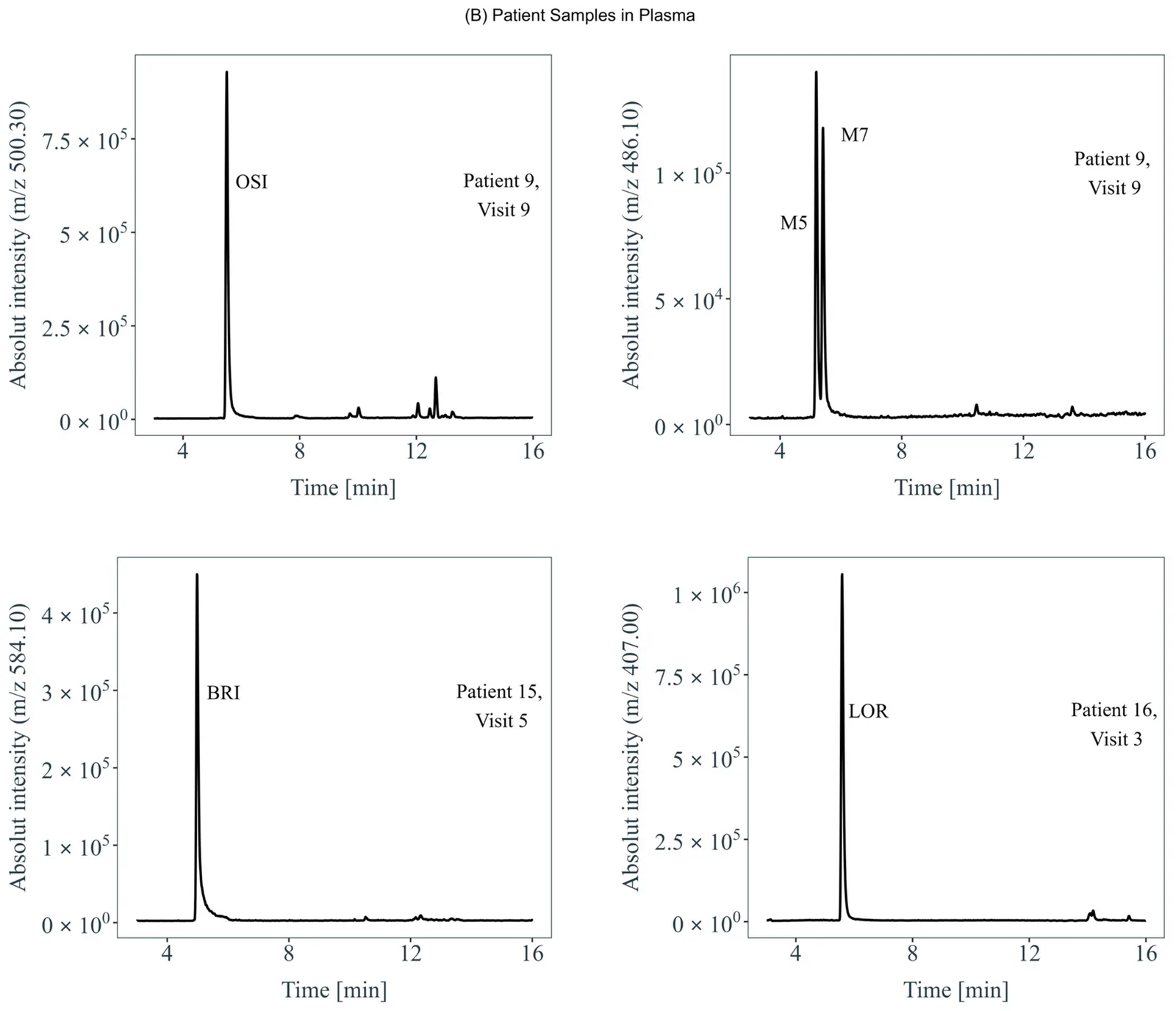
Chromatograms of patient samples for OSI, OSI-M5/M7, LOR and BRI in (**A**) VAMS^®^ and (**B**) plasma.

A total of 100 plasma and 94 VAMS^®^ samples from 34 patients with NSCLC and one patient with DTC were analyzed, resulting in 90 paired samples consisting of a plasma and VAMS^®^ concentration. One sample pair for BRI was not taken consecutively but with a 50 min gap. Four samples were below the LLOQ: one LOR plasma and one VAMS^®^ sample and two OSI plasma samples and one OSI VAMS^®^ sample. The associated metabolite concentrations were also below the LLOQ. The measured plasma and VAMS^®^ concentrations cover a large range. When the concentration is expected to exceed the calibration range, it is diluted accordingly (Table 4). Typical chromatograms are depicted in Figure 4 and Figure S1.

In most cases, plasma trough concentrations have been associated with either or both efficacy and toxicity. Only eight plasma samples were taken at the time of the expected trough (±2 h), of which two were BRI and six were OSI samples. To our knowledge no specific threshold for BRI has been defined, as no significant elevation of adverse events was found when the trough concentration was elevated [3,17]. OSI’s exposure correlates with efficacy and toxicity, leading to a therapeutic window at the trough of 211 to 259 µg/L [5,18]. Two samples meet the efficacy threshold, and none exceed the toxicity threshold. Ishikawa et al. proposed toxicity thresholds for the area under the curve (AUC) over 24 h for OSI and its metabolites combining TDM sampling with population pharmacokinetic (popPK) modeling to predict the AUC [6]. In the case of LOR, concentrations above 400 µg/L at any timepoint correlate with more severe ADR [19]. Out of the 17 measured LOR concentrations, six exceed this threshold, increasing the risk of grade ≥ 3 ADR. The patients not meeting the requirements need closer monitoring for their safety and to ensure the success of the treatment.

When samples are not taken at the expected trough, an interpretation of the results is difficult. To estimate these trough concentrations an approach comparable to the method of combining conventional TDM with popPK modeling by Ishikawa et al. and Mc Laughlin et al. could be implemented [6,20].

**Figure 5:**
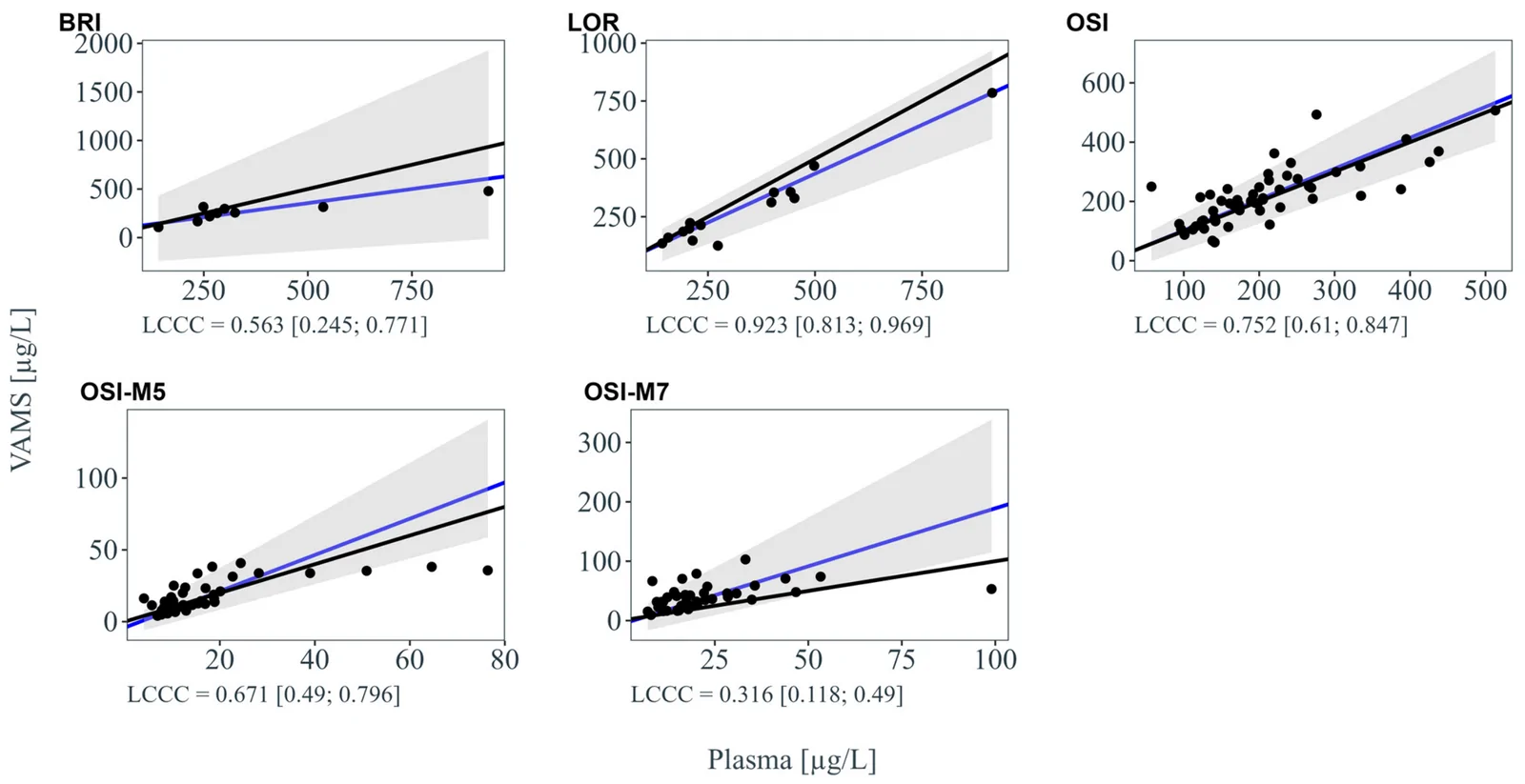
Passing Bablok regression of VAMS^®^ versus plasma concentrations for analytes with at least nine paired samples with Lins’s concordance correlation coefficient for agreement (LCCC [95% confidence interval]). The individual measurements are shown as black dots. The black line represents the line of identity (*y* = *x*), the blue line the Passing Bablok regression and the shaded grey area the 95% confidence interval of the Passing Bablok regression.

To establish a TDM with VAMS^®^ the respective concentrations need to be either transferable between VAMS^®^ and plasma samples or a conversion factor needs to be applied since the TDM targets are defined as plasma concentrations. A Passing Bablok regression was performed for analytes with at least nine paired samples (Figure 5).

For LOR and OSI, the VAMS^®^ concentration showed good agreement with the plasma concentrations, as the Passing Bablok regression lines are close to the line of identity, indicating that no conversion factor is required. In contrast, the regression results for OSI-M5 and OSI-M7 were strongly influenced by a small number of samples with high plasma concentrations, for which the corresponding VAMS^®^ concentrations deviated substantially. Although the passing Bablok regression could also be performed for BRI, only nine paired samples were available, resulting in a wide 95% confidence interval (CI) and consequently greater uncertainty in the estimated regression parameters.

### 3.10. Hematocrit Influence on VAMS^®^-to-Plasma-Ratio

The observed hematocrit in patients ranged from 30 to 45%. For DAB and TRA, there were two paired samples available, so no confidence interval can be calculated and therefore an in vivo hematocrit effect cannot be ruled out. The respective 95%-CI of the slope obtained from the linear regression of ALE (95%-CI: −0.175, 0.149), ALE-M4 (95%-CI: −0.310, 0.346), BRI (95%-CI: −0.092, 0.109), LOR (95%-CI: −0.053, 0.012), OSI (95%-CI: −0.030, 0.078), OSI-M5 (95%-CI: −0.065, 0.082) and OSI-M7 (95%-CI: −0.068, 0.206) included zero, so no significant hematocrit effect on the VAMS^®^-to-plasma-ratio was observed within the studied hematocrit (Figure S2).

## 4. Discussion

When establishing a TDM workflow, the bioanalytical method to determine the concentration of the respective drugs is essential and needs to be robust. Previous studies on monitoring TKIs in patients with metastasized renal cell carcinoma demonstrated that HPLC-MS is well suited to address the needed reliability [21]. Henrikson et al. also concluded that the simultaneous determination of multiple TKIs is appropriate for TDM, as these agents may be co-administered or switched during the course of treatment [22].

There are LC-MS/MS methods utilizing VAMS^®^ published for ALE, ALE-M4, DAB, LEN, OSI and TRA. The method described here also shows feasibility of the detection with an SQ mass spectrometer along with on-line solid phase extraction (SPE). The use of an SQ is limited by its selectivity in complex matrices like whole blood and plasma, which is successfully addressed by integrating the above-mentioned SPE. This extraction method also allows a significant reduction in time and a simpler workflow during sample preparation, as the evaporation for purification described by Meertens et al. and Zimmermann et al. is not necessary. The shorter and less elaborate sample preparation procedure facilitates the implementation of routine TDM in this study [9,11].

Using an SQ (LC-MS) entails the problem of sensitivity, which is compensated for by an increased injection volume of 200 µL for the VAMS^®^ samples. Meertens et al. [9] and Zimmermann et al. [11] injected 2 and 40 µL, respectively, which enabled them to cover a large concentration range. However, this method here is still suitable for TDM across the expected concentration range in patients since dilution integrity was demonstrated for all analytes. The large injection volume to counterbalance the small sample volume indirectly increases the risk of ion suppression and enhancement due to more matrix components being injected as well. The high ion enhancement in the VAMS^®^ samples limits the use of the results since these measurements are subject to a greater degree of uncertainty. A patient individual factor between VAMS^®^ and plasma could allow using the results for TDM purposes. Protein precipitation during sample preparation and the on-line SPE—which are both commonly applied procedures for the preparation of samples containing complex biological matrices—reduce the amount of matrix reaching the detector, resulting in only minor matrix peaks in blank samples [23,24].

The sensitivity and selectivity is the reason that TDM is usually carried out using LC-MS/MS, allowing for an LLOQ of even a few ng/L [23,24]. Admittedly, a very low LLOQ has advantages when monitoring drug concentrations over extended periods after administration or following very low doses. However, when monitoring patients in steady state during a standard dosing interval of 12 or 24 h, the expected concentrations are comparatively high. This is also reflected in the proposed TDM targets, where concentrations are supposed be below 259 µg/L for OSI or below 88 µg/L for LEN at their expected trough level [3]. To monitor these patients and assess the drug concentration for TDM, there is no need to determine concentrations below the LLOQ achieved using LC-MS. The selectivity of a triple quad (TQ, LC-MS/MS) has the advantage of selecting a qualifier and a quantifier to reduce the impact of the matrix effect [23]. The ion ratio of the qualifier and quantifier and the fragmentation are unique, which allows for a distinct differentiation between the analyte and matrix compounds or co-administered drugs. To make full use of this high selectivity, an elaborate tuning process is necessary during method development—which is redundant when using an SQ and therefore streamlines the process of integrating new analytes with an SQ in comparison to a TQ method. Costs represent an important consideration in TDM implementation. The lower acquisition cost of an SQ instrument may enable more laboratories to establish local TDM capabilities. Sample preparation for SQ-based analysis can be slightly more complex compared with TQ systems as additional purification steps may be required to achieve sufficient analytical performance. However, run times can be reduced when coupling a TQ instead of an SQ due to its enhanced selectivity and ability to distinguish coeluting analytes based on their unique fragmentation patterns. This improves not only the turnover rate but also minimizes the use of solvents and therefore results in lower day-to-day operating costs. In routine laboratories with a high volume of samples, costs and time are crucial factors, so using a TQ should be considered. Nevertheless, in laboratories with a few samples per week the use of an SQ presents an alternative due to easier handling with sufficiently robust results for TDM.

The carry-over for some analytes is very high. This behavior was also observed by Stern et al. [25] and explained by the trapping method exploited by the HTLC column. However, when comparing the absolute carry-over to measured and expected concentrations within the therapeutic window, the carry-over for ALE, ALE-M4, BRI and OSI is deemed acceptable as the influence on the sample concentration is below 10%. The influence on OSI-M5 and OSI-M7 concentrations may exceed 10% in the case of low concentrations, so the carry-over precludes the randomization of samples. As far as the TDM results are concerned, this implies that most interpretations will therefore be minimally affected and only samples with very low concentrations may require reanalysis with extended washing phases. The samples should be analyzed according to their anticipated increasing concentration levels while alternating with samples containing other analytes or acetonitrile injections. The needle-washing solution consists of 70% methanol, which provides good solubility for the investigated analytes and is well suited for cleaning of the needle. To minimize carry-over, the trap column was flushed with a methanol/isopropanol mixture containing formic acid after each injection. The use of isotopic labeled internal standards helps to reduce the impact of the carry-over on the result, as they behave similar to the analyte.

To our knowledge, there are currently no methods published to determine BRI, LOR, OSI-M5 or OSI-M7 in capillary blood using VAMS^®^. For OSI and its metabolites, there is a validated method for the determination utilizing the hemaPEN^®^. The results reported by Venkatesh et al. are consistent with the results obtained by us with respect to stability, robustness and reproducibility. Their determination by UPLC-MS/MS allows for shorter run times and covering a comparatively narrower concentration range (1–270 µg/L) [26]. The sampling timepoints in the current study are not limited to the expected trough level immediately before the next dose, which carries the risk of sampling at peak OSI concentrations that may exceed this validated concentration range. The method described here is well suited to quantify high OSI concentrations, as the ULOQ is 500 µg/L.

Capillary blood sampling using VAMS^®^ has been reported to be less dependent on hematocrit [15,27] compared with other whole-blood sampling techniques. Verougstraete and Stove evaluated eight TKIs and showed that VAMS^®^ is a suitable tool for monitoring TKI concentrations, with limited impact from physiological hematocrit variations [10].

A hematocrit effect was found in vitro for DAB and OSI by Zimmermann et al. [11]; however, only samples from one patient taking DAB were included. The method published by Venkatesh et al. did not find an in vitro hematocrit effect for OSI or its metabolites. Paired samples of 15 patients consisting of a capillary blood and venous plasma sample confirmed this finding [26], which aligns well with the observations reported in the present study.

Liu et al. reported comparable estimates of secondary pharmacokinetic parameters (exposure and maximal concentration) based on capillary blood and venous plasma samples of ALE and ALE-M4, although the capillary blood sampling technique was not further specified [28]. This suggests that hematocrit-related matrix differences did not affect the PK analysis significantly. Meertens et al. investigated the in vitro hematocrit effect of ALE and ALE-M4 using VAMS^®^ and applied their method to 10 patient samples, verifying their results [9]. The hematocrit of the included patients from the present study did not cover the full range that can be expected in patients. However, the results are consistent with the results obtained by Meertens et al.

## 5. Conclusions

A robust HPLC-MS method for TDM was developed and validated for the simultaneous quantification of multiple TKIs and relevant metabolites in plasma and capillary blood using the VAMS^®^ technology. Relevant acceptance criteria of bioanalytical guidelines by EMA and FDA were met for both approaches, so it can be concluded that TDM can be performed using VAMS^®^. Further samples need to be included to confirm the independence of the VAMS^®^-to-plasma ratio from the hematocrit and to analyze other influencing factors. This is important to allow for direct interpretation of the VAMS^®^ concentrations in the future.

## Figures and Tables

**Table 1:** Calibration range and quality control levels per analyte.

Analyte	Range [µg/L]	QC Level [µg/L]		
		LQC	MQC	HQC
ALE	1.95–500	6	200	400
ALE-M4	1.95–500	6	200	400
BRI	15.6–500	40	200	400
DAB	15.6–500	40	200	400
LEN	15.6–500	40	200	400
LOR	1.95–500	6	200	400
OSI	1.95–500	6	200	400
OSI-M5	0.977–250	3	100	200
OSI-M7	0.977–250	3	100	200
TRA	3.95–500	20	200	400

**Table 2:** Mean recovery (%CV) and mean matrix effect (%CV) in plasma and VAMS^®^.

Analyte	Level	Plasma (*n* = 3)		VAMS (*n* = 3)	
		Recovery [%]	Matrix Effect [%]	Recovery [%]	Matrix Effect [%]
ALE	LQC	94.3 (3.4)	88.4 (3.9)	90.4 (7.4)	124 (9.3)
	MQC	101 (3.1)	80.2 (0.4)	88.9 (6.4)	158 (2.4)
	HQC	101 (1.7)	80.3 (7.1)	95.8 (1.9)	148 (3.8)
ALE-M4	LQC	93.2 (4.8)	99.2 (4.7)	95.4 (2.7)	151 (2.7)
	MQC	101 (1.6)	84.6 (3.4)	94.5 (7.1)	140 (4.6)
	HQC	105 (2.6)	80.4 (1.8)	96.0 (2.1)	137 (1.1)
BRI	LQC	89.5 (3.0)	125 (9.1)	87.2 (8.3)	218 (8.7)
	MQC	88.5 (3.8)	86.5 (4.9)	99.9 (2.0)	200 (2.1)
	HQC	94.5 (1.0)	70.9 (2.9)	106 (7.6)	153 (4.9)
DAB	LQC	101 (3.4)	96.4 (8.1)	93.0 (10.1)	106 (15.0)
	MQC	96.8 (8.9)	105 (3.1)	76.3 (4.3)	141 (2.9)
	HQC	96.5 (5.2)	114 (5.0)	93.7 (13.9)	115 (9.4)
LEN	LQC	97.0 (2.6)	114 (4.0)	95.8 (4.7)	123 (3.2)
	MQC	94.3 (5.3)	133 (2.3)	94.4 (6.7)	127 (3.5)
	HQC	98.8 (1.5)	127 (1.5)	97.0 (1.9)	118 (1.5)
LOR	LQC	83.7 (2.9)	92.5 (6.2)	80.9 (9.0)	172 (7.8)
	MQC	93.2 (4.6)	69.8 (3.5)	100 (9.7)	135 (2.9)
	HQC	101 (4.3)	61.7 (2.2)	99.1 (1.1)	130 (3.5)
OSI	LQC	91.9 (4.1)	105 (8.4)	64.7 (4.8)	154 (6.3)
	MQC	98.1 (4.2)	103 (4.7)	78.1 (7.0)	133 (6.9)
	HQC	96.7 (3.4)	106 (4.4)	83.3 (4.6)	128 (3.9)
OSI-M5	LQC	85.4 (7.1)	114 (7.3)	72.2 (10.2)	187 (6.3)
	MQC	90.7 (2.0)	114 (6.2)	80.3 (2.2)	131 (4.6)
	HQC	95.0 (2.2)	103 (1.8)	83.6 (5.4)	125 (5.8)
OSI-M7	LQC	88.0 (9.9)	153 (4.3)	56.6 (12.4)	216 (1.4)
	MQC	104 (3.4)	123 (6.6)	62.6 (2.3)	159 (4.4)
	HQC	101 (2.3)	127 (6.3)	72.8 (13.8)	134 (3.9)
TRA	LQC	102 (6.5)	86.9 (7.7)	88.7 * (17.0)	109 (3.9)
	MQC	98.1 (6.4)	104 (1.0)	84.7 (7.1)	159 (3.5)
	HQC	97.4 (0.7)	117 (4.6)	102 (4.3)	140 (6.3)

* One VAMS^®^ sample was underfilled. Mean recovery (*n* = 2) is 97.0%.

**Table 3:** Between-run and within-run comparison of plasma and VAMS^®^.

Analyte	QC-Level	Plasma				VAMS^®^			
		Within Run		Between Run		Within Run		Between Run	
		Accuracy [%]	CV [%]	Accuracy [%]	CV [%]	Accuracy [%]	CV [%]	Accuracy [%]	CV [%]
ALE	LLOQ	113	3.44	112	5.30	90.5	5.93	97.7	9.54
	LQC	100	3.59	106	4.16	89.4	2.37	101	7.98
	MQC	103	1.57	103	6.29	96.0	6.59	97.7	7.48
	HQC	98.9	2.25	98.0	9.19	93.8	3.19	94.2	4.98
ALE-M4	LLOQ	107	3.36	109	8.52	101	8.31	105	7.84
	LQC	105	1.62	105	8.07	98.0	8.51	102	8.09
	MQC	95.9	2.65	98.7	6.83	99.2	5.85	102	7.61
	HQC	95.5	2.62	99.1	4.04	101	3.08	96.8	8.11
BRI	LLOQ	108	2.83	106	5.14	97.3	8.04	106	7.52
	LQC	94.8	3.71	103	2.11	100	12.1	92.3	4.67
	MQC	109	3.40	105	6.93	105	4.71	100	9.32
	HQC	105	4.44	104	4.49	108	3.41	101	9.92
DAB	LLOQ	97.1	5.31	96.7	3.87	105	4.18	104	4.21
	LQC	94.3	5.41	93.7	5.92	101	7.58	103	8.58
	MQC	99.3	2.02	95.2	5.13	108	5.05	102	9.83
	HQC	98.1	2.37	95.8	8.50	109	3.71	104	8.63
LEN	LLOQ	93.5	8.76	92.9	6.98	103	6.70	102	8.52
	LQC	97.5	6.48	94.0	7.50	92.5	5.34	98.4	5.93
	MQC	99.0	3.88	90.8	4.21	106	3.56	106	7.06
	HQC	99.2	2.30	90.0	4.27	111	3.02	102	7.87
LOR	LLOQ	99.4	6.33	107	7.52	107	4.54	101	8.44
	LQC	94.3	4.50	110	3.72	104	5.33	191	5.40
	MQC	92.2	2.36	90.5	2.36	111	2.51	97.5	7.15
	HQC	90.2	2.41	97.9	6.98	106	4.92	99.6	5.59
OSI	LLOQ	99.3	5.51	100	3.59	101	9.77	99.6	11.35
	LQC	104	2.61	105	4.12	102	5.38	103	8.59
	MQC	89.8	3.85	106	4.74	104	6.29	102	9.47
	HQC	90.8	2.77	104	6.53	110	1.43	97.9	9.66
OSI-M5	LLOQ	104	3.44	110	2.69	101	9.72	103	7.00
	LQC	102	4.48	107	6.97	105	7.97	103	4.82
	MQC	89.9	3.03	103	7.61	108	5.76	96.3	4.82
	HQC	86.6	1.24	103	3.41	111	2.27	94.0	6.21
OSI-M7	LLOQ	107	5.93	108	7.27	102	5.77	107	9.06
	LQC	105	2.93	99.5	5.22	106	6.80	100	8.47
	MQC	103	2.93	97.5	7.37	106	3.86	97.2	8.88
	HQC	98.6	2.43	101	5.68	105	4.24	95.8	6.87
TRA	LLOQ	92.4	2.45	97.4	4.48	103	6.86	106	7.83
	LQC	93.9	2.35	93.4	4.85	96.2	2.37	98.5	6.98
	MQC	98.7	8.74	100	5.33	105	4.89	100	6.52
	HQC	99.6	8.77	99.4	5.80	109	0.87	103	9.56

**Table 4:** Number of included patients and summary of analyzed patient samples.

Analyte	Number of Plasma Samples	Number of VAMS^®^ Samples	Number of Patients	Number of Samples per Patients	Range of Measured Plasma Concentration [µg/L]	Range of Measured VAMS^®^ Concentration [µg/L]
ALE	7	5	5	1–2	62.7–755	144–950
ALE-M4	7	5	5	1–2	31.1–314	90–594
BRI	9	9	3	1–6	141–934	107–479
DAB	2	2	1	2	1220–1430	809–866
LEN	2	2	1	2	23–131	29.1–58.3
LOR	17	16	7	1–6	2.25–914	67.2–785
OSI	61	58	19	1–9	57.2–513	2.42–507
OSI-M5	61	46	19	1–9	4.02–76.4	4.09–40.8
OSI-M7	61	46	19	1–9	7.08–99	9.44–103
TRA	2	2	1	2	9.48–12.1	33.4–33.5

## Data Availability

The original contributions presented in this study are included in the article. Further inquiries can be directed to the corresponding author.

## Supplementary Materials

The following supporting information can be downloaded at https://www.mdpi.com/article/10.3390/pharmaceutics18080923/s1. Table S1. Summary of linearity of calibration curves per analyte and matrix (*n* = 6). Table S2. Summary of selectivity per analyte and matrix (*n* = 6). Table S3. Summary of carry-over per analyte in plasma (*n* = 3). Table S4. Summary of reinjection reproducibility per analyte in plasma (*n* = 5). Table S5. Summary of dilution integrity in plasma (*n* = 5). Figure S1. (A) Chromatograms of patient samples in VAMS^®^ for DAB, TRA, ALE, ALE-M4 and LEN. (B) Chromatograms of patient samples in plasma for DAB, TRA, ALE, ALE-M4 and LEN. Figure S2. Linear regression of the VAMS^®^-to-plasma ratio as a function of hematocrit for each analyte. Data points below the LLOQ were excluded for this analysis to avoid bias in the regression analysis.

## Abbreviations

The following abbreviations are used in this manuscript:

ADR	Adverse Drug Reaction
AGA	Actionable Genetic Alteration
ALE	Alectinib
AUC	Area Under the Curve
BRI	Brigatinib
CI	Confidence Interval
DAB	Dabrafenib
DBS	Dried Blood Spot
DTC	Differentiated Thyroid Carcinoma
EDTA-WB	EDTA-Whole Blood
EMA	European Medicines Agency
FDA	Food and Drug Administration
HPLC-MS	High Performance Liquid Chromatography-Mass Spectrometry
HQC	High Concentration Quality Control Sample
LEN	Lenvatinib
LLOQ	Lower Limit of Quantification
LQC	Low Concentration Quality Control Sample
LOR	Lorlatinib
MQC	Mid Concentration Quality Control
NSCLC	Non-Small Cell Lung Cancer
OSI	Osimertinib
popPK	Population Pharmacokinetic (Modeling)
SQ	Single Quadrupole
TDM	Therapeutic Drug Monitoring
TKI	Tyrosine Kinase Inhibitor
TQ	Triple Quadrupole
TRA	Trametinib
ULOQ	Upper Limit of Quantification
VAMS	Volumetric Absorptive Microsampling
