# Supplementary material for: HPLC-MS Quantification of Multiple Tyrosine Kinase Inhibitors in Patients with Solid Tumors: Method Validation and Clinical Application
Source: Pharmaceutics. 2026 Jul 27;18(8):923. doi: 10.3390/pharmaceutics18080923 (PMC13516218; doi:10.3390/pharmaceutics18080923)
Supplement: Supplementary file 1 [file pharmaceutics-18-00923-s001.zip › pharmaceutics-4390934-supplementary.pdf]

Supplementary Materials: HPLC-MS Quantification of Multiple Tyrosine Kinase Inhibitors in Patients with Solid Tumors: Method Validation and Clinical Application

Juliane Staudinger [1], Marcel Kemper [2], Carolin Krekeler [2], Lea Reitnauer [2], Annalen Bleckmann [2,3] and Georg Hempel [1,*]

1 Department of Clinical Pharmacy, Institute of Pharmaceutical and Medicinal Chemistry, University Muenster, 48149 Muenster, Germany; j.staudinger@uni-muenster.de
2 Medical Clinic A, Hematology, Oncology, Hemostaseology and Pneumology, University Hospital
Muenster, 48149 Muenster, Germany; marcel.kemper@ukmuenster.de (M.K.); carolin.krekeler@ukmuenster.de (C.K.); leaelisabeth.reitnauer@ukmuenster.de (L.R.); annalen.bleckmann@ukmuenster.de (A.B.)
3 West German Cancer Center, University Hospital Muenster, 48149 Muenster, Germany
* Correspondence: georg.hempel@uni-muenster.de

Table S1. Summary of Linearity of Calibration Curves per Analyte and Matrix (*n*=*6*)

Analyte	Plasma Coefficient of determination (R^2)	VAMS Coefficient of determination (R^2)
ALE	0.995	0.993
ALE-M4	0.996	0.994
BRI	0.996	0.996
DAB	0.995	0.996
LEN	0.996	0.994
LOR	0.994	0.993
OSI	0.996	0.994
OSI-M5	0.995	0.994
OSI-M7	0.995	0.995
TRA	0.995	0.996

Table S2. Summary of Selectivity per Analyte and Matrix (*n*=*6*)

Analyte	Plasma [%]	VAMS [%]
ALE	2.13	< 1
ALE-M4	5.55	< 1
BRI	18.3	11.6
DAB	< 1	< 1
LEN	< 1	< 1
LOR	< 1	< 1
OSI	17.2	19.5
OSI-M5	11.3	17.7
OSI-M7	10.4	19.6
TRA	3.90	< 1
$^{2}H_{4}$-ALE-M4	< 1	< 1
D_9-DAB	< 1	< 1
$^{13}C_1$-D_3-OSI	< 1	4.6
$^{13}C_6$-TRA	2.9	< 1

Table S3. Summary of Carry-over per Analyte in Plasma (*n*=3)

Stoff	Run 1 [%]	Run 2 [%]	Run 3 [%]
ALE	44	33	27
ALE-M4	32	12	2
BRI	127	56	36
DAB	3	< 1	< 1
LEN	< 1	< 1	< 1
LOR	5.7	< 1	< 1
OSI	205	65	31
OSI-M5	105	33	13
OSI-M7	273	75	33
TRA	15	< 1	< 1

Table S4. Summary of Reinjection Reproducibility per Analyte in Plasma (*n*=5)

Analyte	LQC		MQC		HQC	
	Accuracy [%]	CV [%]	Accuracy [%]	CV [%]	Accuracy [%]	CV [%]
ALE	90.4	4.18	96.2	3.85	95.3	2.42
ALE-M4	96.6	6.20	100	5.08	96.1	2.95
BRI	91.4	4.06	98.6	6.56	90.3	3.10
DAB	93.5	5.31	94.1	7.77	88.8	2.58
LEN	90.5	5.38	95.3	3.00	88.9	2.33
LOR	95.1	9.71	89.4	3.21	88.2	1.91
OSI	91.1	5.62	90.0	4.59	89.4	1.81
OSI-M5	92.1	3.77	88.6	3.47	87.3	2.19
OSI-M7	92.8	5.88	95.8	4.02	90.9	2.72
TRA	90.2	5.42	90.7	1.54	104	4.49

Table S5. Summary of Dilution Integrity in Plasma (*n*=5)

Analyte	1:1		1:3	
	Accuracy [%]	CV [%]	Accuracy [%]	CV [%]
ALE	97.5	6.00	90.3	3.24
ALE-M4	99.9	6.06	88.5	3.40
BRI	96.4	3.43	90.8	4.95
DAB	98.2	1.70	94.6	3.90
LEN	90.9	3.04	94.6	8.53
LOR	95.6	2.05	91.1	8.07
OSI	96.8	4.11	91.9	5.54
OSI-M5	98.3	4.48	90.6	4.97
OSI-M7	100	3.41	88.9	3.91
TRA	103	6.47	96.9	1.94

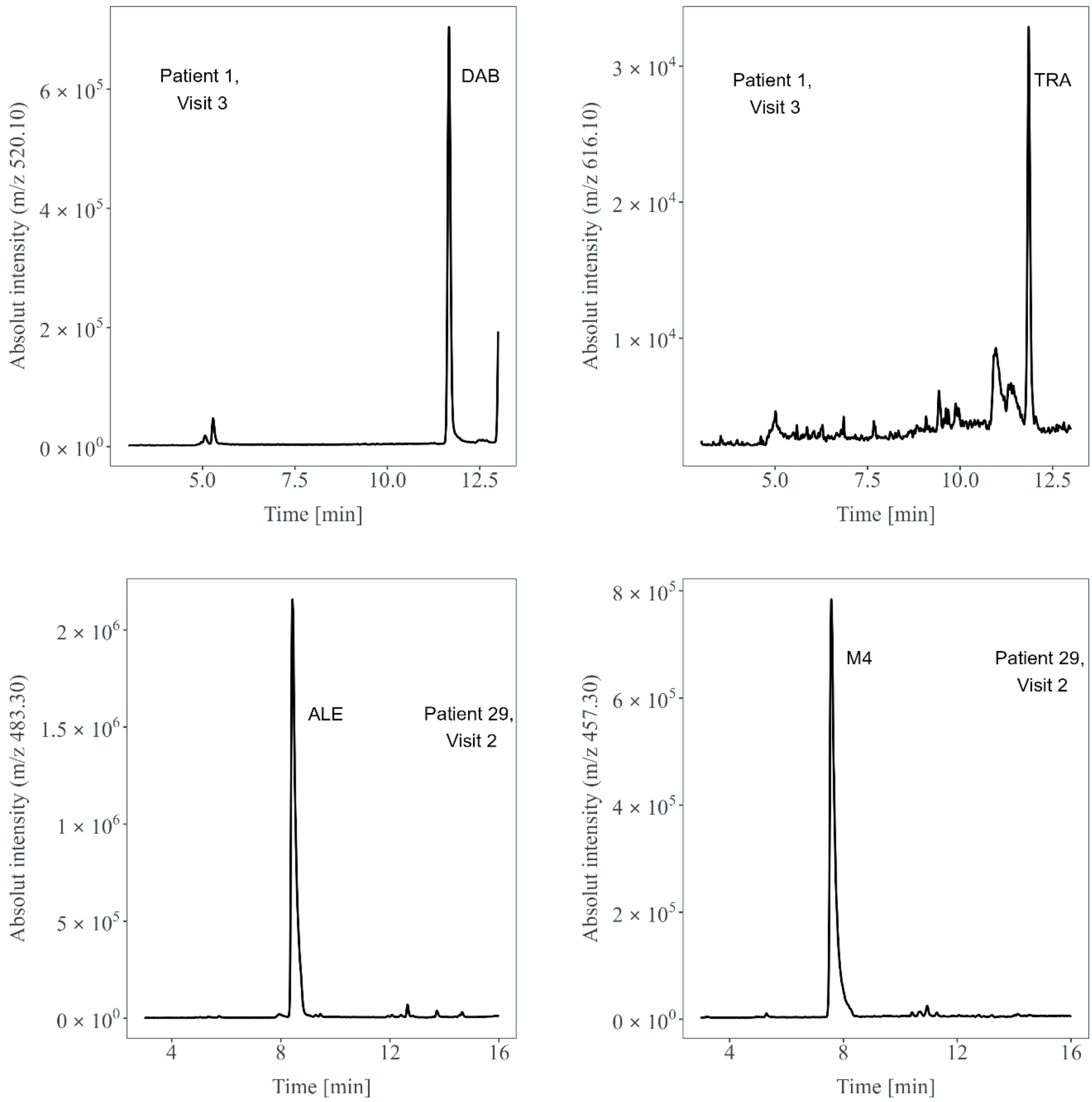

Figure S1A. Chromatograms of Patient Samples in VAMS® for DAB, TRA, ALE, ALE-M4 and LEN

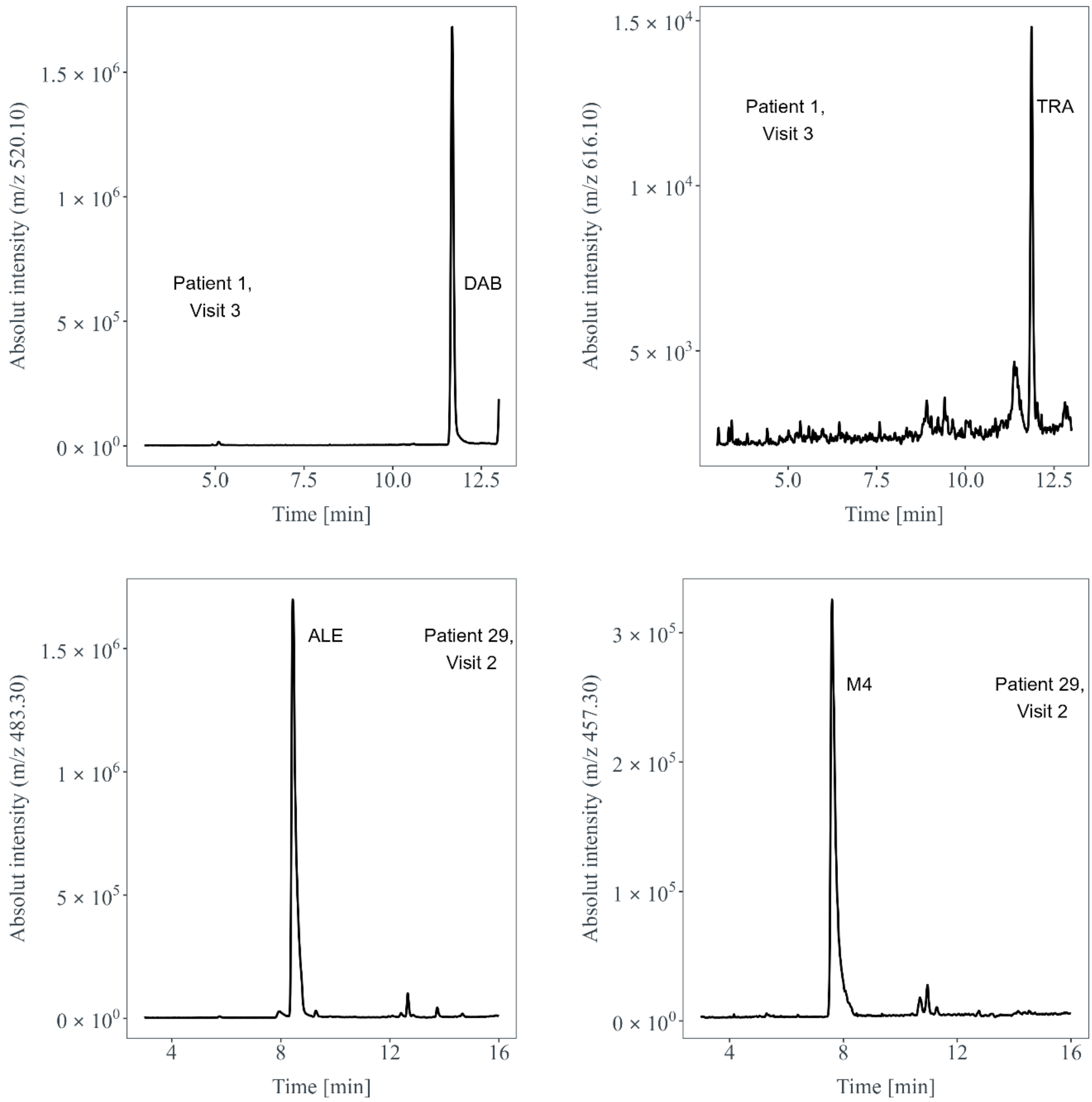

Figure S1B. Chromatograms of Patient Samples in Plasma for DAB, TRA, ALE, ALE-M4 and LEN

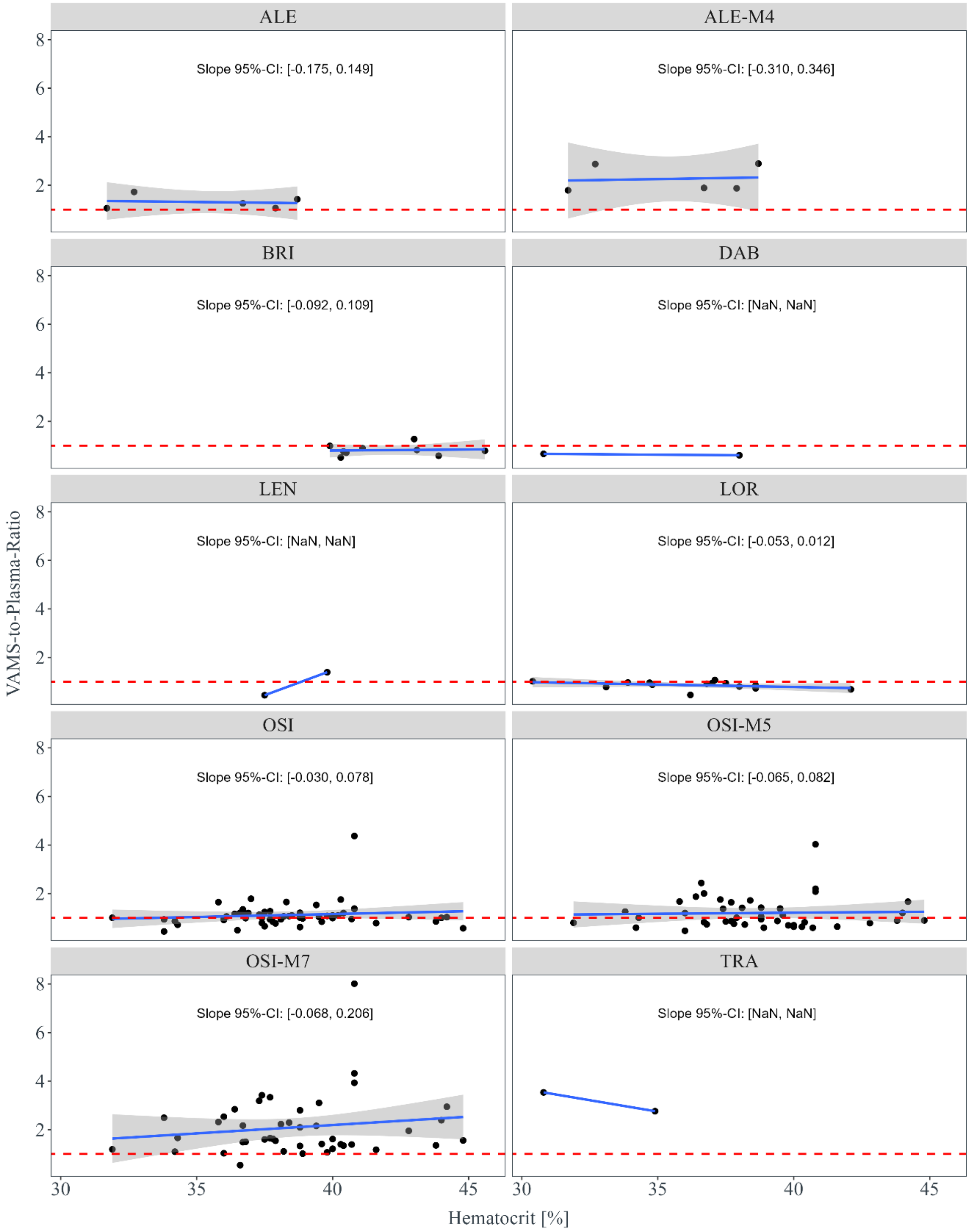

Figure S2. Linear regression of the VAMS®-to-Plasma-Ratio as a function of hematocrit for each analyte. Data points below the LLOQ were excluded for this analysis to avoid bias in the regression analysis.
